# Red Blood Cell Distribution Width (RDW) Is a Potential Surrogate Biomarker of Inflammation in Patients With Achalasia: A Cohort Study

**DOI:** 10.7759/cureus.97877

**Published:** 2025-11-26

**Authors:** Diana Aguilar-León, Dulce P Méndez-Hernández, Miguel Moreno-Fuentes, Enrique Coss-Adame, Luis R Valdovinos-García, Miguel A Valdovinos, Andric Perez-Ortiz, Janette Furuzawa-Carballeda, Gonzalo Torres-Villalobos

**Affiliations:** 1 Experimental Surgery, Instituto Nacional de Ciencias Médicas y Nutrición Salvador Zubirán, Mexico City, MEX; 2 Surgery and Experimental Surgery, Instituto Nacional de Ciencias Médicas y Nutrición Salvador Zubirán, Mexico City, MEX; 3 Gastroenterology, Instituto Nacional de Ciencias Médicas y Nutrición Salvador Zubirán, Mexico City, MEX; 4 Gastroenterology, Instituto Nacional de Ciencias Médicas y Nutrición Salvador Zubirán, Mexico, MEX; 5 Surgery, ABC Medical Center, Mexico City, MEX; 6 School of Medicine, Universidad Panamericana, Mexico City, MEX

**Keywords:** achalasia, gastroesophageal reflux disease, inflammatory disease, integrated relaxation pressure (irp), red blood cell distribution width (rdw)

## Abstract

Background

Current studies demonstrate that red blood cell distribution width (RDW) is a potential surrogate biomarker of inflammation. This study aims to compare the RDW and RDW/lymphocytes ratio in patients with achalasia who underwent five-year postoperative follow-up with those in the gastroesophageal reflux disease (GERD) and healthy donor groups, and to evaluate their clinical relevance in relation to integrated relaxation pressure (IRP), sex, inflammation and the type of achalasia (fibrosis).

Materials and methods

This was a retrospective study with a five-year follow-up. One hundred sixty-one patients with achalasia, 161 patients with GERD and 500 healthy donors (HD) were included and followed up for five years. The achalasia and GERD patient groups were matched with the HD control group based on demographic characteristics and laboratory variables. The diagnoses of achalasia and GERD were made using high-resolution esophageal manometry, upper gastrointestinal endoscopy, barium esophagography, and 24-hour pH monitoring. For the achalasia group, the correlation between RDW and clinical characteristics, including Eckardt score, Eat Assessment Tool (EAT-10) score, the GERD-Related Quality of Life (GERD-HRQL) questionnaire score, achalasia type, sex, comorbidities, and integrated relaxation pressure, was evaluated using logistic regression analysis.

Results

The RDW values at baseline differed significantly between patients (achalasia and those with GERD) and HD (*P*<0.001). During follow-up, the achalasia group had substantially higher RDW values ​​than the GERD group (*P*=0.031). Patients with achalasia experienced a sustained increase in RDW during follow-up compared to their baseline values (All: *P*=0.010; type I: *P*=0.006; type II: *P*<0.001; females: *P*=0.003; males: *P*=0.948).

Conclusion

These results underscore the significance of RDW as an inflammatory marker, demonstrating notable temporal variation depending on sex and type of achalasia. This finding contrasts sharply with the stability of the RDW observed in patients with GERD.

## Introduction

Achalasia is a primary chronic esophageal motility disorder characterized by aperistalsis and failure of the lower esophageal sphincter to relax. The pathophysiology of achalasia seems to involve an autoimmune process, including an inflammatory component [1-3]. The characteristic distribution of cells and cytokines that promotes an inflammatory microenvironment in the lower esophageal sphincter tissue supports this theory [4,5].

Flow cytometry, immunofluorescence, and RNA sequencing analyses have revealed that patients with achalasia suffer from systemic chronic low-grade inflammation, characterized by dysregulated immune cells and mediators associated with disease duration [4,6-8]. Low-grade inflammation has been proposed as an underlying pathophysiological mechanism linking risk factors or metabolic disorders (e.g., oxidative stress, obesity, diabetes, and dyslipidemia) to an increased risk of chronic degenerative diseases as well as a common pathogenic denominator in age-related diseases. Nonetheless, in most patients, the complete blood count (CBC) remains unaltered, or any alterations are related to other underlying pathologies [9]. Additionally, no simple and clinically relevant biomarkers have been identified for the diagnosis and follow-up of patients with achalasia. This highlights the need to enhance research on the utility of clinical parameters obtained through routine laboratory tests.

In this vein, beyond its widely established role in treating anemia, red blood cell distribution width (RDW) has been considered a promising biomarker of inflammation. Red blood cells (RBCs) are non-nucleated cells characterized by their typical oval biconcave shape, diameter of 6-8 μm, and thickness of approximately 2 μm. The average volume of RBCs ranges from 80 to 100 femtoliters (fL), but different physiological and pathological conditions may increase the degree of anisocytosis. RDW is a quantitative measure of variation in the size of circulating RBCs and is receiving increasing interest as a diagnostic and prognostic marker in a vast array of human disorders, including autoimmune and inflammatory diseases, functional bowel conditions, COVID-19, various types of cancer, and multiple hospital admissions in subjects with chronic conditions [10,11]. RDW was calculated automatically by hematological analyzers by dividing the standard deviation (SD) of the mean corpuscular volume (MCV) by the MCV and multiplying by 100 to yield a percentage value. An RDW value below the reference range was considered clinically irrelevant.

In contrast, an increased RDW value indicates a more pronounced difference in the size of RBCs, which can be attributed to the presence of both smaller and larger RBCs. An elevated RDW typically results from either increased or ineffective production of RBCs, or excessive fragmentation or destruction of RBCs [12]. These studies suggest that altered RDW, indicating a higher level of systemic inflammation and oxidative stress, could be associated with worse outcomes and more severe disease in multiple pathologies [13]. Therefore, the purpose of this study was to explore whether RDW and RDW/lymphocytes ratio are altered in achalasia, whether these alterations persists over time, whether it differs between types of achalasia (fibrosis), and whether it serves as a surrogate marker for sex, as well as to explore associations with IRP and inflammatory indices and if they differ among patients with gastroesophageal reflux disease (GERD), and healthy donors (HD) [14,15].

This article was previously posted to the Research Square preprint server on February 14, 2025. Diana Aguilar-León, Dulce P. Méndez-Hernández, Miguel Moreno-Fuentes et al. Role of red blood cell distribution width in the evaluation and follow-up of patients with achalasia, https://doi.org/10.21203/rs.3.rs-5983523/v1

## Materials and methods

Participants

This retrospective nested cohort study was conducted at the Instituto Nacional de Ciencias Médicas y Nutrición Salvador Zubirán, a tertiary referral center, from 2013 to 2024. This study adhered to the Strengthening the Reporting of Observational Studies in Epidemiology (STROBE) statement, along with references to STROBE and the broader Enhancing the Quality and Transparency of Health Research (EQUATOR) guidelines.

One hundred sixty-one patients with achalasia, 161 with GERD (according to Lyon 2.0 criteria for the objective diagnosis of GERD, including Los Angeles grade B, C, or D esophagitis, or with an acid exposure time (AET) greater than 6.0% in 24-hour studies), and 500 HD (one case for every three healthy volunteers) were included. The demographic characteristics were matched to those of the control group. The diagnoses of achalasia and GERD were made using high-resolution esophageal manometry (HRM), upper gastrointestinal endoscopy, barium esophagogram, and 24-hour pH monitoring.

Inclusion and exclusion criteria

Due to the rarity of the pathology, we did not determine the sample size, so recruitment was conducted through convenience sampling. Only patients aged 18 years and older with complete five-year follow-up data were included in the study. Patients with the following diagnoses were excluded: pregnancy; paraneoplastic achalasia, Chagas disease (esophageal involvement is quite rare in Mexico and the United States, in which the disease primarily presents with cardiac rather than esophageal involvement); esophageal stricture; scleroderma; gastric cancer; esophageal cancer; peptic stricture; other esophageal motility disorders; severe hematologic, renal, or hepatic disease; and patients on anticoagulant, aspirin, or steroid therapy. Patients' clinical records were carefully reviewed according to a pre-established protocol. The following data were collected retrospectively for each study participant from the hospital's medical records: demographic features, clinical characteristics, type of achalasia, family history of autoimmunity, current diagnosis of organ or systemic autoimmunity (17%-19% of patients had autoimmune comorbidity and 68% had a family history of autoimmunity [3]), and neurological diseases (epilepsy, migraine, Parkinson's disease, amyotrophic lateral sclerosis, and frontotemporal degeneration). When a comorbid autoimmune or neurological diagnosis was found in patients with achalasia, all relevant data (i.e., date of diagnosis, presenting symptoms, clinical and laboratory confirmatory test results, and treatment administered) were recorded. Finally, chronic inflammatory conditions (asthma, allergic rhinitis, gout, and rosacea) were recorded in the achalasia group. Patients were not evaluated for vitamin B2 deficiency. The complete blood count (CBC) parameters used in the study were the latest laboratory findings recorded five years before and after surgical intervention.

For comparison, 500 healthy donors (HD) matched by sex who were within ±six years of the average age of the patients were recruited from a blood bank as controls. None of the included controls had known cardiovascular, metabolic, inflammatory, or neoplastic diseases. Each healthy volunteer underwent a panel of tests for infections such as hepatitis B, hepatitis C, HIV, human T-lymphotropic virus (HTLV) I and II, syphilis, Chagas disease, and brucellosis. A blood type and complete blood count were also determined, along with a check of the donor's blood pressure, height, weight, and temperature. Donors must not have had tattoos, piercings, or acupuncture in the past 12 months, nor have undergone any surgery in the past six months. Demographic and laboratory data were also collected for this group.

Esophageal high-resolution manometry

Esophageal high-resolution manometry (HRM) was performed in patients with achalasia before referral for surgery. A solid-state HRM probe with 36 circumferential sensors (Medtronic, Minneapolis, MN, USA) was used. The patient was placed in a sitting position at 45°, and stationary esophageal HRM was performed. After a 12-hour fasting period, the probe was inserted transnasally until it passed the esophagogastric junction, and its position was assessed visually on a computer screen. Ten water swallows of 5 ml were provided, with a 30-second interval between each. Analyses were performed using Manoview 2.0 (Medtronic^©^ Manoscan, Medtronic, Minneapolis, MN, USA). Patients were classified into three groups according to the latest Chicago classification v4.011,12: (a) type I achalasia (without pressurization within the esophageal body), (b) type II (with >20% pan-pressurization), and (c) type III (spastic). Two gastroenterologists (EC-A and MAV), as well as experts in esophageal HRM, independently performed the classification; however, inter-observer agreement was not measured.

High-resolution manometry data analysis

The analysis was performed using ManoView software (Given Imaging, Yokneam, Israel). Four critical metrics of HRM - distal contractile integral (DCI), basal EGJ pressure, intrabolus pressure (IBP), and integrated relaxation pressure (IRP)- were used to assess the pressure motor activity of the esophageal body and EGJ. Achalasia was defined as an IRP >15 mmHg and aperistalsis. Type I achalasia was defined as the absence of peristalsis with no compartmentalization of the intrabolus pressure. Type II achalasia was defined as panesophageal pressurization in at least 20% of swallows, as indicated by a 30-mmHg isobaric contour. Type III achalasia is characterized by premature contractions with a shortened distal latency (<4.5 s) in at least 20% of the swallows.

Laboratory information

All CBC analyses were performed using an automatic hematological analyzer (Beckman Coulter DxH 800 Hematology Analyzer, Beckman Coulter, Inc., Brea, CA, USA). Hemoglobin (Hb), red blood cell distribution width - coefficient of variation (RDW-CV; reference range: 12.6%-17.3%), white blood cell (WBC) count, and neutrophil, lymphocyte, monocyte, eosinophil, and platelet counts were obtained. Blood samples were collected in tubes containing dipotassium ethylenediaminetetraacetic acid (EDTA).

Clinical evaluation

Following a diagnosis of achalasia, patients were asked to complete three standardized and validated questionnaires prospectively: the Eckardt symptom score, the EAT-10 (Eat Assessment Tool), and the GERD-Related Quality of Life (GERD-HRQL) questionnaire. These instruments are designed to assess the frequency and severity of symptoms associated with esophageal diseases [16-19]. The IRP from manometry was collected in patients with achalasia to evaluate disease severity [20].

Statistical analysis

Descriptive statistics were computed. The Shapiro-Wilk test was used to evaluate data normality. Since the variables did not meet the assumption of normality, non-parametric tests were applied. Continuous parameters were expressed as mean±standard error of the mean (SEM) or standard deviation (SD). Continuous variables, including age, body mass index (BMI), disease evolution, questionnaires, neutrophil-to-lymphocyte ratio (NLR), RDW, RDW/lymphocyte ratio, platelets, hemoglobin, and IRP, were analyzed using Kruskal-Wallis one-way analysis of variance (ANOVA) on ranks. All pairwise multiple comparison procedures were performed using the Dunn or Holm-Sidak method. Qualitative parameters are expressed as numbers and percentages. Categorical variables were compared using the *𝜒*^2^ test or Fisher's exact test. Pearson's correlation coefficient was used to assess the correlation between hematologic indices, clinical questionnaire scores, and HRM parameters in the distribution of RDW values among all cases and controls, as well as between the different subtypes of achalasia. Linear regression analyses were also performed to explore the relationship between RDW and sex, the prevalence and incidence of autoimmune and inflammatory diseases, and IRP, which are closely associated with symptomatic severity. To adjust for potential confounders, multivariate linear and logistic regression analyses were conducted, reporting adjusted coefficients and odds ratios with 95% confidence intervals. No formal a priori sample size calculation was performed, as this was a retrospective nested cohort study; instead, all available eligible patients during the study period were included, which provided sufficient power for the primary comparisons. A P-value <0.05 was considered statistically significant for all analyses. These were performed using JASP (Jeffrey's Amazing Statistics Program) software version 0.19 for Mac OS Sonoma 14.5 (University of Amsterdam, Amsterdam, Netherlands).

Ethical considerations

This study was approved by the Research Committee and Research Ethics Committee of the National Institute of the Instituto Nacional de Ciencias Médicas y Nutrición Salvador Zubirán, Reference Number: 1522, and CONBIOÉTICA-09-CEI-011-20160627 in accordance with the principles outlined in the 1989 Declaration of Helsinki. All patients participated voluntarily, and only those who provided written informed consent were included in this study.

## Results

Demographic and clinical characteristics

**Figure 1:**
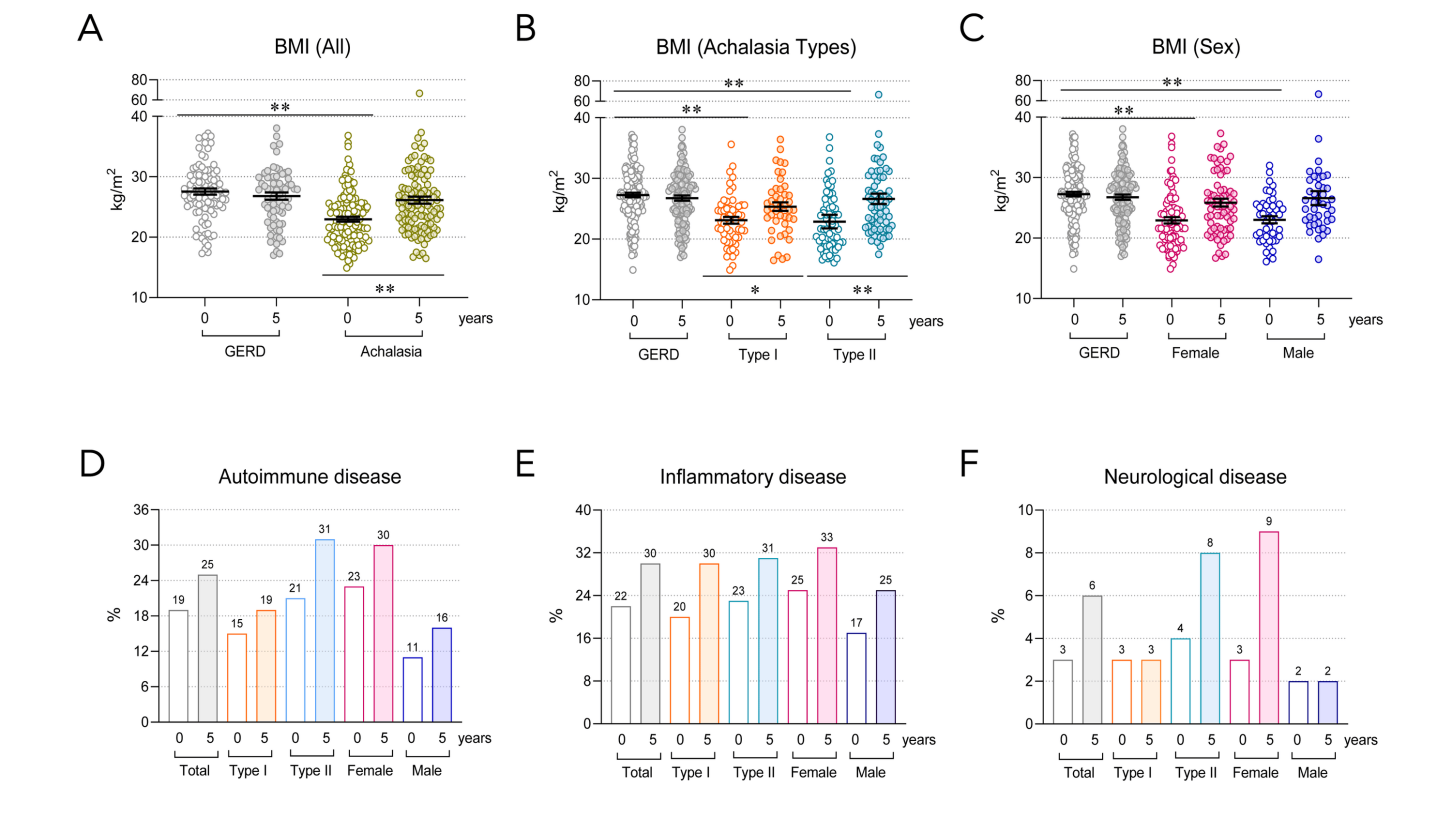
Clinical characteristics of the patients at baseline and follow-up. Body mass index (BMI) in (A) all achalasia, gastroesophageal reflux disease (GERD), and healthy donor (HD) groups; (B) type I and II achalasia, and (C) female and male. The results are expressed as the mean±SEM. *P<0.05 and **P<0.001. Comorbidities of patients with achalasia (D) autoimmune disease, (E) inflammatory disease, (F) neurological disease. The results are expressed as a percentage. GERD (n=161); achalasia (n=161); Type I achalasia (n=60); Type II achalasia (n=101); Female patients with achalasia (n=107); male patients with achalasia (n=54).

Sixty-six percent of patients with achalasia were women, with a mean age of 48 years and a preoperative mean body mass index of 23 kg/m^2^ (Figure 1A-C). At diagnosis, 99% of the patients had dysphagia, 92% had regurgitation, and 88% had weight loss. The prevalence of autoimmune, inflammatory, and neurological comorbidities was 19% (Figure 1D), 22% (Figure 1E), and 3% (Figure 1F), respectively, in patients with achalasia (Table 1). None of the patients had paneoplastic achalasia.

**Table 1:** Demographic, clinical, and laboratory variables at baseline

	Healthy Donors (n=500)	GERD (n=161)	Achalasia Total (n=161)	Achalasia Male (n=54)	Achalasia Female (n=107)	Type I Achalasia (n=60)	Type II Achalasia (n=101)
Demographics							
Age (years), mean±SD Median (Range)	36.6±11.7 36 (18-63)	49.9±11.1 50 (18-66)	47.9±15.0 49 (22-64)	47.2±14.6 48 (22-76)	48.3±15.3 50 (24-86)	48.5±14.5 50 (24-83)	47.2±15.4 48 (22-86)
Sex, Female (%) Male (%)	315 (63) 185 (37)	107 (66) 54 (34)	107 (66) 54 (34)	0 (0) 54 (100)	107 (100) 0 (0)	31 (51) 29 (48)	74 (75) 24 (24)
Disease evolution (mo), mean ± SD Median (Range)	ND	ND	30.3±41.5 13 (1-288)	33.0±53.1 13 (2-288)	28.9±34.5 13 (1-156)	39.3±55.3 18 (1-288)	25.2±30.0 12 (1-156)
Body mass index (kg/m^2^) mean ± SD Median (Range)	ND	26.8±5.1 28 (17-46)	23.0±4.5 23 (15-37)	23.0±3.8 23 (16-32)	22.9±4.7 22 (15-37)	23.1±4.2 23 (15-36)	22.9±4.7 22 (16-37)
Clinical							
Dysphagia (%)	ND	ND	160 (99)	53 (98)	107 (100)	59 (98)	98 (100)
Regurgitation (%)	ND	ND	148 (92)	48 (88)	100 (93)	56 (93)	91 (92)
Weight loss (%)	ND	ND	142 (88)	48 (88)	94 (87)	55 (91)	85 (86)
Heartburn (%)	ND	ND	104 (64.5)	29 (53)	75 (70)	39 (65)	65 (66)
Autoimmune disease (%)	ND	ND	30 (19)	6 (11)	24 (23)	9 (15)	21 (21)
Inflammatory disease (%)	ND	ND	35 (22)	9 (17)	26 (25)	12 (20)	23 (23)
Neurological disease (%)	ND	ND	6 (3)	2 (3)	4 (3)	2 (3)	4 (4)
Questionnaires							
GERD-HQRL, mean±SD Median (Range)	ND	ND	24.3±12.7 22 (0-48)	22.3±13.2 22 (2-47)	25.4±12.4 23 (0-48)	25.6±13.7 22 (2-47)	23.6±12.0 23 (0-48)
EAT-10, mean±SD Median (Range)	ND	ND	29.9±9.0 34 (1–43)	28.0±9.8 30 (1-40)	31.0±8.4 35 (4-43)	30.0±8.3 32 (1-40)	29.9±9.6 34 (4-43)
ECKARDT, mean±SD Median (Range)	ND	ND	9.1±2.2 9 (2 – 12)	8.9±2.5 9 (2-12)	9.2±2.1 10 (4-12)	9.3±2.4 10 (2-12)	9.0±2.1 9 (3-12)
Laboratory variables							
Hemoglobin (g/dL), mean±SD Median (Range)	15.3±1.1 15.3 (13.3-18.6)	14.4±1.8 14.5 (5.7-19.6)	14.6±1.5 14.5 (10.0 -18.2)	15.7±1.2 15.8 (13.6-18.2)	14.0±1.3 14.2 (10.0-17.5)	14.8±1.6 14.7 (10.0-17.9)	14.5±1.5 14.5 (10.1-18.2)
RDW (%), mean±SD Median (Range)	10.4±0.5 10.4 (8.9-13.7)	13.6±0.7 13.5 (12.8- 15.0)	13.6±0.7 13.5 (12.6 -14.4)	13.7±0.8 13.5 (12.5-16.0)	13.8±1.2 13.5 (12.3-20.5)	13.9±1.2 13.6 (12.3-20.5)	13.7±1.0 13.6 (12.5-17.8)
Platelets 10^3/μL, mean±SD Median (Range)	275±52 271 (144-460)	263±60 258 (108-499)	242±58 240 (135-427)	222±41 223 (150-326)	250±64 244 (135-427)	238±53 231 (145-392)	244±61 244 (135-427)
Leukocytes 10^3/μL, mean±SD Median (Range)	7.0±1.5 6.9 (3.6-11.2)	6.7±2.4 6.5 (2.8-23.1)	6.54±1.9 6.3 (2.9-12.4)	6.6±1.7 6.6 (3.6-11.4)	6.5±1.9 6.1 (2.9-12.4)	6.4±2.0 6.1 (2.9-11.4)	6.6±1.8 6.6 (3.5-12.4)
Lymphocytes (%), mean±SD Median (Range)	33.4±7.1 32.8 (5.8-55.8)	30.5±9.1 29.7 (2.5-50.0)	29.6±9.2 29.6 (5.5- 54.9)	29.3±9.7 29.6 (10.9-54.9)	29.7±9.0 29.6 (5.5-50.6)	29.5±9.4 29.7 (10.9-47.0)	29.6±9.3 29.6 (5.5-54.9)
Monocytes (%), mean±SD Median (Range)	7.4±1.4 7.3 (3.5-14.2)	7.6±2.5 7.4 (2.1-28.0)	7.1 ± 1.8 7.0 (1.0-13.6)	7.5±1.4 7.6 (4.7-11.9)	6.9-2.0 6.8 (1.0-13.6)	7.7±1.7 7.6 (4.4-13.6)	6.8±1.8 6.8 (1.0-13.0)
Eosinophils (%), mean±SD Median (Range)	2.3±1.8 1.8 (0.0-22.6)	2.1±1.6 1.9 (0-13.0)	2.4±2.2 1.9 (0.0-13.0)	2.7±2.4 2.2 (0.1-13.0)	2.2±2.0 1.7 (0.0- 13.0)	2.7±2.7 2.1 (0.1-13.0)	2.2±1.8 1.8 (0.0-12.0)
Basophils (%), mean±SD Median (Range)	1.0±0.6 1.0 (0.2-12.0)	0.64±0.4 0.60 (0- 13.0)	0.6±0.4 0.6 (0.0-5.0)	0.5±0.3 0.6 (0.0-2.0)	0.6±0.5 0.6 (0.0-5.0)	0.6±0.4 0.6 (0.0-2.0)	0.6±0.5 0.6 (0.0-5.0)
Neutrophils (%), mean±SD Median (Range)	56.0±7.7 56.2 (31.7-77.0)	58.9±10.5 58.7 (31-91.4)	59.1±11.2 59.4 (6.1-92.4)	59.6±10.8 59.4 (33.0-79.1)	58.8±11.4 59.4 (6.1-92.4)	58.7±10.4 58.6 (37.0-78.5)	59.3±11.9 59.4 (6.1-92.4)
CRP (mg/dL), mean±SD Median (Range)	ND	0.77±1.2 0.36 (0.09-3.6)	0.57±1.0 0.23 (0.02- 4.2)	0.4±0.6 0.1 (0.02-3.2)	0.3±0.7 0.1 (0.02-4.7)	0.3±0.6 0.1 (0.02-3.2)	0.4±0.7 0.1 (0.02-4.7)

During the five-year postoperative follow-up of patients with achalasia, the body mass index increased by 12%, dysphagia decreased by 80%, regurgitation decreased by 82%, and weight loss increased by 83%. The prevalence of autoimmune comorbidities rose from 19% to 25%, inflammatory comorbidities increased from 22% to 30%, and neurological comorbidities rose from 3% to 6% (Table 2).

**Table 2:** Demographic, clinical, and laboratory variables at follow-up (60 months)

	GERD (n=161)	Achalasia Total (n=161)	Achalasia Male (n=54)	Achalasia Female (n=107)	Type I Achalasia (n=60)	Type II Achalasia (n=101)
Demographics						
Age (years), mean ± SD Median (Range)	47±12.1 48 (22-88)	48.3±15.5 49.0 (19-84)	47.5±16.1 47.5 (19-84)	48.7±15.2 49 (21-84)	48.3±15.7 43.5 (19-84)	47.0±15.4 44.0 (21-81)
Body mass index (kg/m^2^) mean ± SD Median (Range)	27.5±5.3 27.4 (17.3-45.7)	26.1±6.3 25.2 (16.5-66.6)	26.6±7.5 25.5 (16.5-66.6)	25.9±5.5 25.2 (16.7-45.3)	25.3±4.7 25.2 (16.5-36.4)	26.6 ±7.1 25.3 (17.5-66.6)
Clinical						
Dysphagia (%)	ND	48 (32)	17 (32)	31 (31)	15 (27)	33 (35)
Regurgitation (%)	ND	27 (17)	11 (20)	16 (16)	11 (20)	16 (16)
Weight loss (%)	ND	15 (15)	6 (15)	9 (14)	8 (18)	6 (11)
Weight gain (%)	ND	71 (73)	27 (71)	44 (74)	30 (68)	41 (78)
Heartburn (%)	ND	37 (24)	16 (30)	21 (21)	14 (25)	23 (24)
Autoimmune disease (%)	ND	41 (25)	9 (16)	32 (30)	11(19)	30 (31)
Inflammatory disease (%)	ND	48 (30)	13 (25)	35 (33)	18 (30)	30 (31)
Neurological disease (%)	ND	10 (6)	2 (3)	9 (9)	2(3)	8 (8)
Questionnaires						
GERD-HQRL, mean±SD Median (Range)	ND	4.3±5.3 3.0 (0-26)	3.5±5.6 1.0 (0 – 26)	4.7±5.1 3.5 (0-23)	3.1±4.8 0.0 (0-18)	5.1±5.6 4.0 (0-26)
EAT-10, mean±SD Median (Range)	ND	2.7±4.4 1.0 (0-24)	2.6±5.0 1.0 (0-24)	2.7±4.1 1.0 (0-19)	2.2±3.6 1.0 (0-14)	3.0±4.9 1.0 (0-24)
Eckardt, mean±SD Median (Range)	ND	1.7±1.5 1.0 (0-8)	1.6±1.6 1.0 (0-8)	1.7±1.4 1.0 (0-0.6)	1.8±1.1 2.0 (0-4)	1.6±1.7 1.0 (0-0.8)
Laboratory variables						
Hemoglobin (g/dL), mean ± SD Median (Range)	14.6±1.8 14.7 (5.7-19.6)	14.0±2.0 14.0 (6.1-18.2)	15.7±1.4 16.0 (12.4-18.2)	13.3±1.9 13.5 (6.1-17.4)	14.2±2.3 14.3 (6.1-18.2)	13.9±1.9 14.0 (6.1-17.9)
RDW (%), mean±SD Median (Range)	13.7±0.8 13.6 (12.7-14.3)	14.7±1.9 13.9 (12.6-26.5)	13.8±1.2 13.5 (12.3-18.2)	15.4±2.8 14.3 (12.3-26.5)	15.2±3.0 14.0 (12.7-26.5)	15.0±2.3 14.2 (12.3- 25.1)
Platelets 10^3 / μL, mean±SD Median (Range)	254±62 255 (108-476)	245±58 242 (128-433)	29±47 230 (150-348)	251±61 249 (128-433)	241±63 242 (129-433)	248±56 243 (128-372)
Leukocytes 10^3 / μL, mean±SD Median (Range)	6.7±2.4 6.5 (2.8-23.1)	6.3±2.3 6.0 (3.0-21.6)	6.8±3.1 6.2 (3.7-21.6)	6.2±2 5.9 (3-13.8)	6.6±2.2 6.4 (3.4-13.8)	6.2±2.4 5.9 (3.0-21.6)
Lymphocytes (%), mean±SD Median (Range)	30.5±9.1 29.7 (2.5-50.0)	29.3±8.8 29.8 (3.0-54.5)	28.3±10.5 30.3 (3.0-54.5)	29.7±8.2 29.7 (7-47.8)	28.7±10.4 30.0 (5.1-54.5)	29.6±8.1 29.4 (3.0-43.1)
Monocytes (%), mean±SD Median (Range)	7.7±2.5 8.0 (3.6-28)	7.6±2.4 7.4 (2.0-22.0)	8.1±1.7 8.0 (4.5-12.4)	7.4± 2.5 7.1 (2.0–22.0)	7.5±2.05 7.7 (2.0-12.2)	7.6±2.6 7.3 (4.0-22.0)
Eosinophils (%), mean±SD Median (Range)	2.1±0 1.9 (0-12.8)	3.2±6.8 2.1 (0.0-75.0)	2.9±2.2 2.4 (0.0-9.2)	3.3±7.9 2.0 (0.0-75)	4.6±11.1 2.3 (0.0-75.0)	2.4±2.2 1.9 (0.0-12.8)
Basophils (%), mean±SD Median (Range)	0.6±0.4 0.6 (0-3.4)	0.6±0.3 0.6 (0.0-2.3)	0.6±0.3 0.6 (0.1-1.2)	0.6±0.3 0.5 (0.0-2.3)	0.6±0.4 0.6 (0.0-2.3)	0.5±0.3 0.5 (0.0-1.6)
Neutrophils (%), mean±SD Median (Range)	57.8±12.5 57.6 (3.6-91.4)	58.4±11.5 58.8 (8.1-90.2)	57.3±13.6 58.9 (8.1-90.2)	58.8±10.7 58.7 (12-85.9)	57.1±15.6 58.7 (8.1-90.2)	59.1±8.7 59.1 (43.0-81.9)

The patients with achalasia experience an increase in body mass index after myotomy

Patients with achalasia, regardless of type and sex, had a significantly lower preoperative body mass index than those in the GERD group (P<0.001). However, this difference disappeared during the follow-up (Figure 1A-C).

The prevalence of autoimmune, inflammatory, and neurological diseases increases in patients with achalasia during a five-year follow-up

The prevalence of autoimmune diseases in the achalasia group was 25%: type I achalasia (19%), type II achalasia (31%), female achalasia (30%), and male achalasia (16%). The most significant increases were observed in type II achalasia and in female patients (Figure 1D).

The prevalence of inflammatory diseases was 30% in the achalasia group, 30% in type I, 31% in type II, 33% in women, and 30% in men. The most significant increase was observed in patients with type I achalasia (Figure 1E).

Finally, neurological diseases (epilepsy, migraine, and Parkinson's disease) increased from 3 to 6% (basal vs. five-year follow-up) in the achalasia group, which was attributed to patients with achalasia type II and female sex (Figure 1F).

Symptom assessment in patients with achalasia pre- and post-myotomy decreases to clinically significant scores

**Figure 2:**
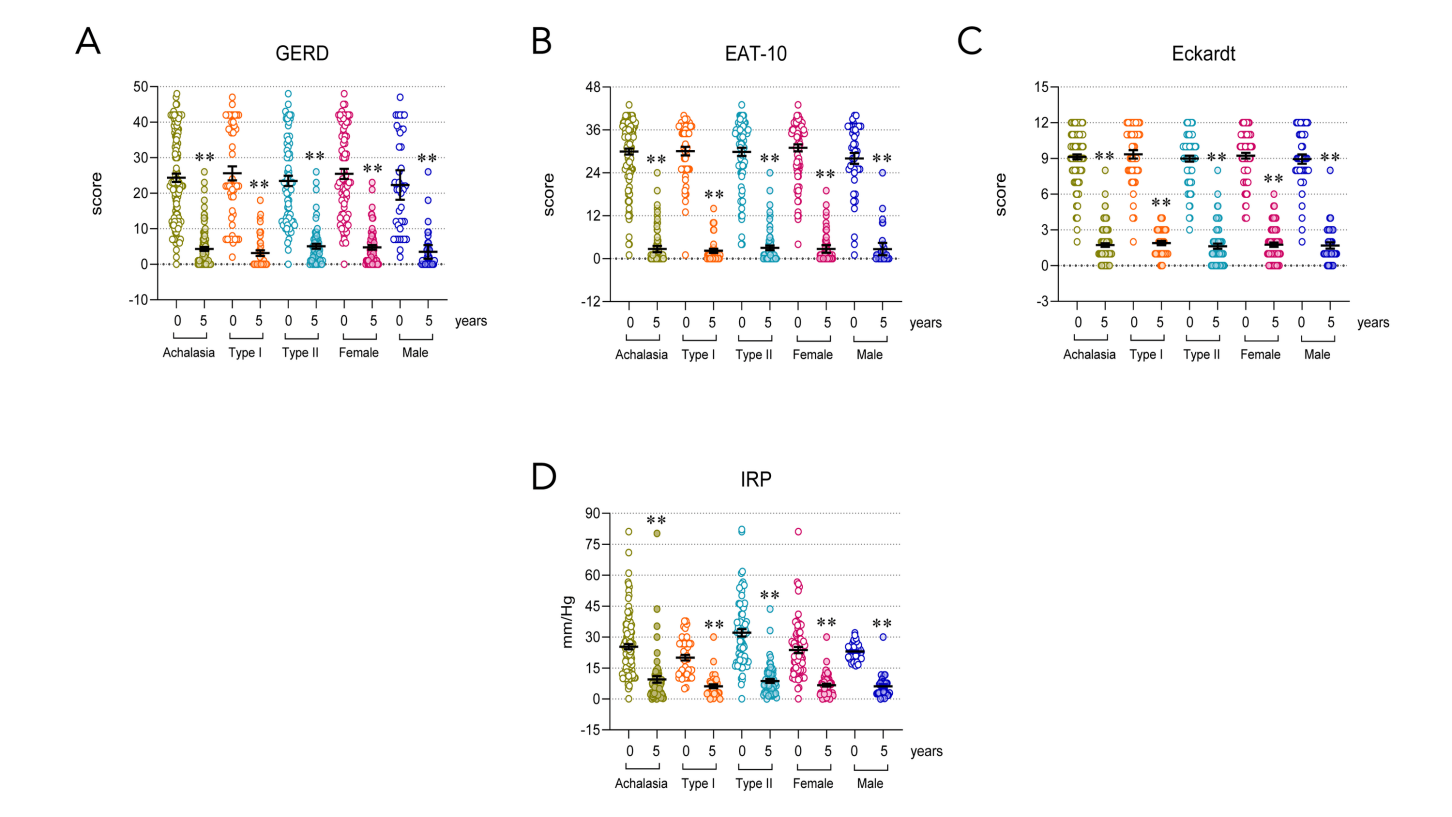
Symptomatic evaluation and IRP of patients at baseline and follow-up (A) GERD questionnaire, (B) EAT-10 questionnaire, (C) Eckardt questionnaire, and (D) IRP in all, type I, type II, female, and male patients with achalasia. Achalasia (n=161); type I achalasia (n=60); type II achalasia (n=101); female patients with achalasia (n=107); male patients with achalasia (n=54). The results are expressed as the mean±SEM. *P<0.05 and **P<0.001. GERD: gastroesophageal reflux disease; EAT-10: Eat Assessment Tool; IRP: integrated relaxation pressure.

According to the international standardized and validated questionnaires, the Eckardt score, used for evaluating symptoms and efficacy of the treatment, the EAT-10 measures swallowing difficulties, and the GERD-HRQL, which measures GERD symptoms, patients with achalasia showed significant improvement at five years post-myotomy compared with baseline (P<0.001, Figure 2A-C).

The integrated relaxation pressure in patients with achalasia during the five-year follow-up is within normal parameters, regardless of the type of achalasia and the patient's sex

Patients with achalasia showed abnormal IRP at baseline. Type II achalasia had a higher IPR than type I achalasia (P<0.001). At the five-year follow-up, regardless of achalasia type or patient sex, the IRP returned to normal parameters after surgical intervention (IRP<15 mmHg; P<0.001, Figure 2D).

The patients with achalasia exhibit alterations in hemoglobin and platelet counts, which persist during follow-up

**Figure 3:**
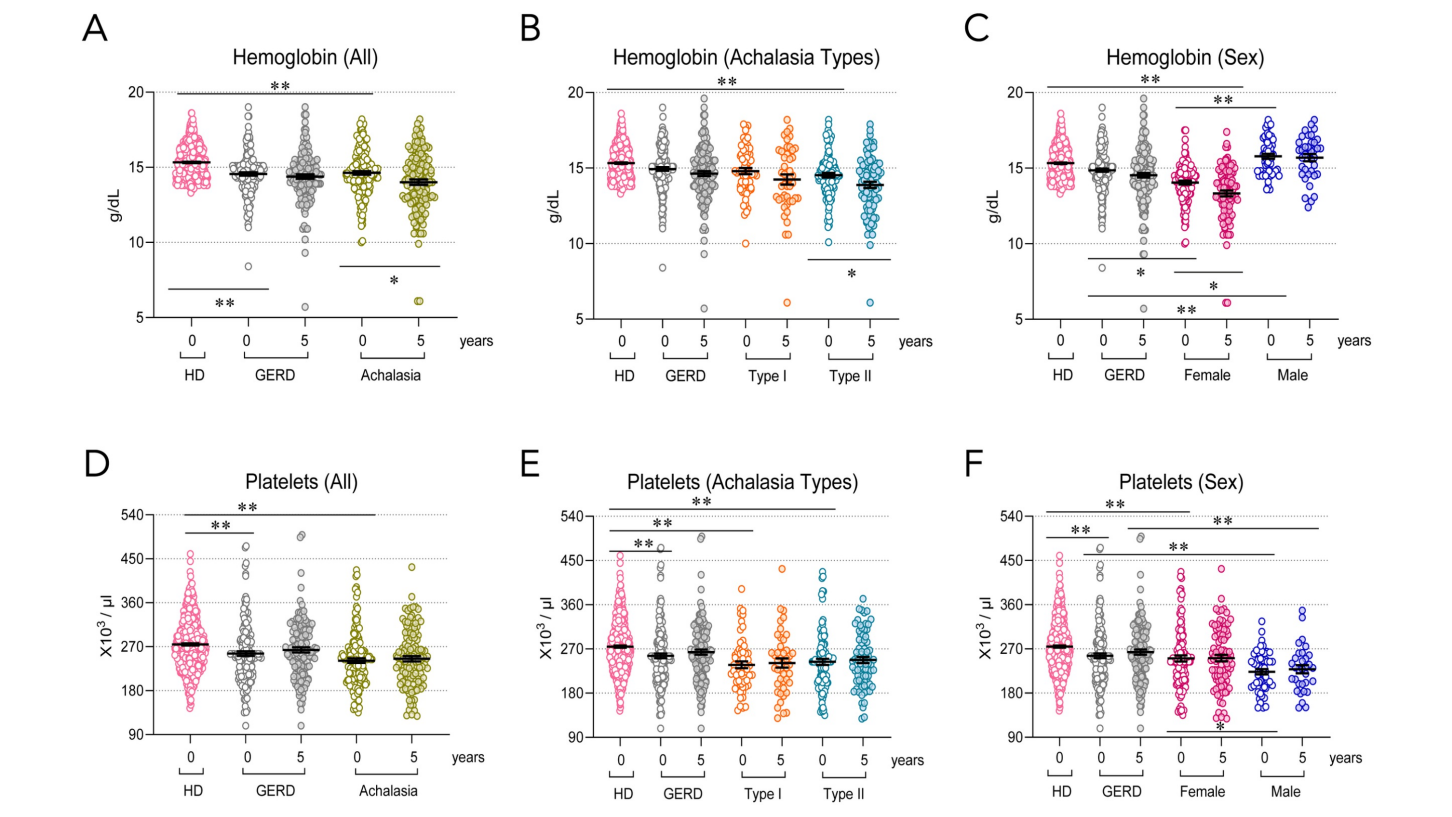
Hematological parameters in patients at baseline and during follow-up. Hemoglobin in (A) controls (HD), all achalasia, and GERD patients; (B) type I and II achalasia, and (C) female and male patients with achalasia. Platelets in (D) all achalasia and GERD patients and controls; (E) type I and II achalasia, and (F) female and male patients with achalasia. HD: Healthy donors (n=500); GERD (n=161); achalasia (n=161); Type I achalasia (n=60); Type II achalasia (n=101); Female patients with achalasia (n=107); Male patients with achalasia (n=54). The results are expressed as the mean ± SEM. *P<0.05 and **P<0.001. GERD: gastroesophageal reflux disease.

The baseline hemoglobin concentration in achalasia and GERD patients was lower than that in healthy individuals (P<0.001; Figure 3A), and it was associated with a decrease in type II achalasia (P<0.001; Figure 3B) and females (P<0.001; Figure 3C), but not in males or type I achalasia. The decrease in hemoglobin level became even more pronounced during follow-up compared to baseline in patients with type II achalasia (P=0.029, Figure 3B) and in female patients (P=0.003, Figure 3C). In men, hemoglobin levels remained within normal parameters (P<0.001; Figure 3C).

The baseline platelet cell numbers in the GERD and achalasia types I and II patient groups, including both male and female patients, were significantly lower than those in the healthy individual group (P<0.001; Figure 3D-F). During follow-up, male patients with achalasia had lower platelet cell counts than those with GERD (P<0.001, Figure 3F).

The patients with achalasia exhibit alterations in RDW, RDW/lymphocyte ratio, and neutrophil-to-lymphocyte ratio, which persist during follow-up

Several hematological indices, such as the neutrophil-to-lymphocyte ratio (NLR), monocyte-to-lymphocyte ratio (MLR), RDW, and platelet-related markers, have been proposed as potential indicators of systemic inflammation. These markers have been studied in autoimmune, cardiovascular, and chronic inflammatory diseases, and their roles in achalasia and response to treatment continue to be investigated.

**Figure 4:**
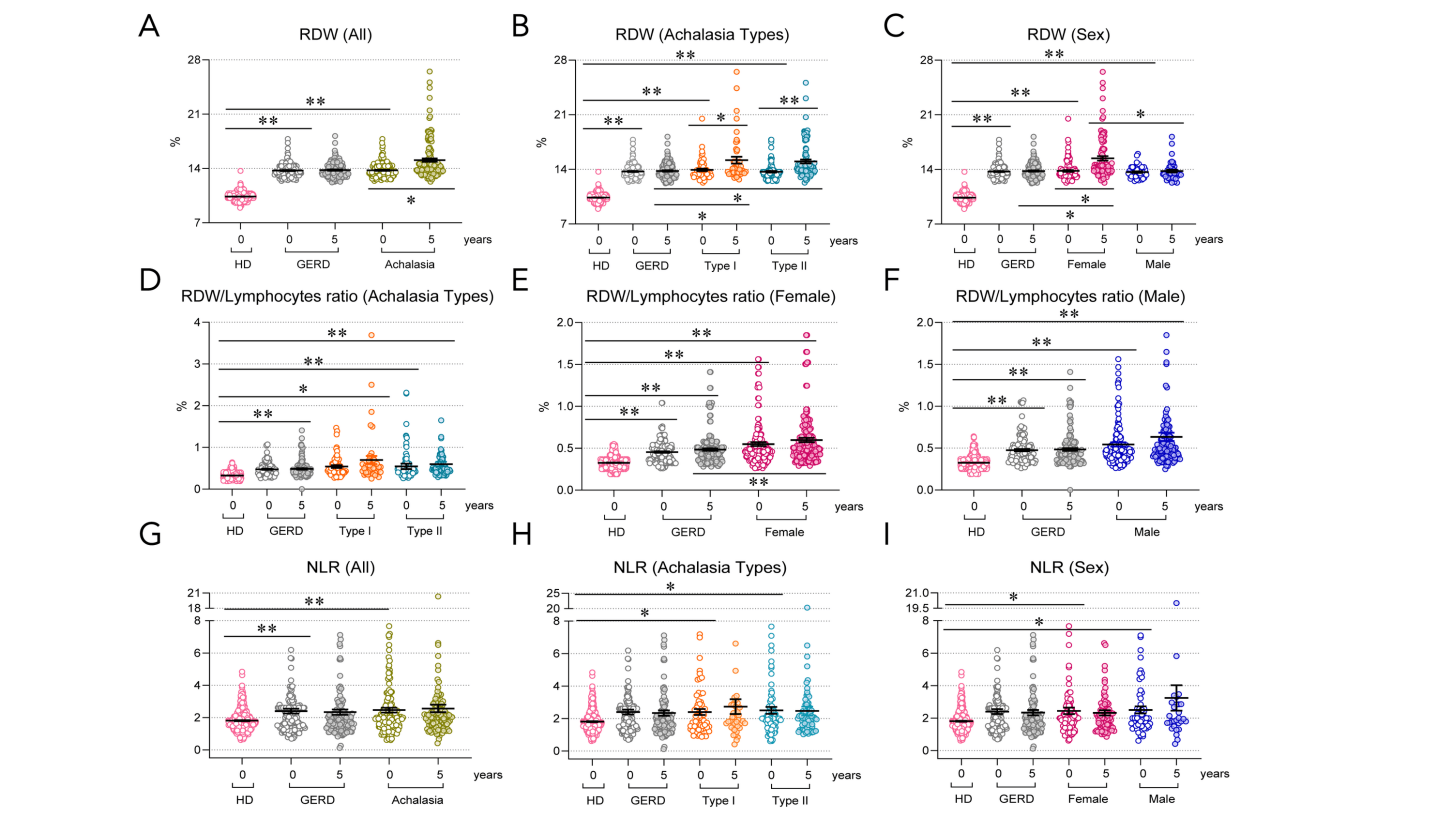
RDW, RDW/lymphocyte ratio, and NLR ratio in patients with achalasia at baseline and during follow-up. RDW in (A) all achalasia and GERD patients and controls at baseline and follow-up; (B) type I and II achalasia, and (C) female and male patients with achalasia. RDW/lymphocytes ratio in (D) types, (E) female, and (F) male patients with achalasia. Neutrophil-to-lymphocyte (NLR) ratio in (G) all achalasia, GERD, and HD groups; (H) type I and II achalasia, and (I) female and male patients with achalasia. HD: Healthy donors (n=500); GERD (n=161); achalasia (n=161); Type I achalasia (n=60); Type II achalasia (n=101); Female patients with achalasia (n=107); male patients with achalasia (n=54). The results are expressed as the mean±SEM. *P<0.05 and **P<0.001. RDW: Red blood cell distribution width; GERD: gastroesophageal reflux disease; HD: healthy donor.

The RDW at baseline was significantly higher in the GERD and achalasia patient groups compared to HD (P<0.001; Figure 4A). Patients with achalasia types I and II (P<0.001; Figure 4B) had a significantly higher erythrocyte distribution width than those with HD (P<0.001; Figure 4C), regardless of gender.

During follow-up, the RDW increased significantly in the achalasia type I (P=0.006; Figure 4B), type II (P<0.001; Figure 4B), and female groups (P=0.006; Figure 4C) compared to their respective baseline values. In the male group, the RDW remained unchanged throughout the follow-up period (Figure 4C). This increase suggests that RDW changes over time in response to the natural evolution of the disease, which affects only females, regardless of the type of achalasia.

In contrast, RDW in patients with GERD showed no significant differences between the time of diagnosis and follow-up, indicating that RDW in this population remained stable.

The RDW/lymphocyte ratio has emerged as a potent diagnostic marker with a strong correlation to fibrosis and substantial predictive value for it. Patients with achalasia types I and II had a significantly increased RDW/lymphocyte ratio compared with HD (P<0.01; Figure 4D). For patients with type II achalasia, the increase in this index at the fifth year of follow-up was 11% compared to baseline. In contrast, for patients with type I achalasia, it was 40%. Subanalysis of the female group with achalasia revealed a significantly higher RDW/lymphocyte ratio compared to the HD and GERD groups at five years of follow-up (P<0.001; Figure 4E). Male patients with achalasia had a significantly higher RDW/lymphocyte ratio than those with HD (P<0.001; Figure 4F). For female patients with achalasia, the increase in this index at the fifth year of follow-up was 9% compared to baseline. In contrast, for male patients, it was 17%.

Achalasia is associated with a higher neutrophil-to-lymphocyte ratio than healthy individuals

Patients with achalasia (P<0.001, Figure 4G), regardless of type (type I, P=0.047; and type II, P=0.005; Figure 4H) and sex (female: P=0.006; and male: P=0.040; Figure 4I), had a higher neutrophil-to-lymphocyte ratio than healthy individuals. The NLR increased slightly during follow-up (Figure 4G-I). No significant differences were observed in the GERD group.

Predictive value of clinical characteristics

A five-year linear regression analysis was conducted to investigate the impact of sex and autoimmune disease on RDW alterations. In patients with achalasia, both sex and autoimmune comorbidities influenced erythrocyte distribution width (Table 3).

**Table 3:** Linear regression

Model Summary- RDW Achalasia at follow-up (%)								
Model	R	R^2^	Adjusted R^2^	RMSE	R^2^ Change	df1	df2	p
Sex: Follow-up								
M_0_	0.000	0.000	0.000	2.552	0.000	0	132	
M_1_	0.213	0.045	0.038	2.503	0.045	1	131	0.014
Note. M_1_ includes sex								
Autoimmune disease: Follow-up								
Model	R	R^2^	Adjusted R^2^	RMSE	R^2^ Change	df1	df2	p
M_0_	0.000	0.000	0.000	2.552	0.000	0	132	
M_1_	0.181	0.033	0.026	2.520	0.033	1	131	0.037
Note. M_1_ includes Autoimmune disease								

However, the strongest association was found between the IRP and RDW in patients with achalasia. It was statistically significant between baseline and follow-up, with a coefficient of determination (R²) of 0.762 and a P value <0.001 (Table 4). The coefficient of IRP was 0.384 (Â±0.020), indicating that a high IRP was associated with a higher RDW. This finding reinforces the relevance of IRP as a tangible parameter for assessing the severity of achalasia, which is closely related to patient symptoms.

**Table 4:** Linear Regression: IRP baseline

Model	R	R^2^	Adjusted R^2^	RMSE	R^2^ Change	df1	df2	p
M_1_	0.873	0.762	0.760	6.753	0.762	1	119	<0.001

## Discussion

RDW is a simple and inexpensive clinical test that can be performed in any laboratory setting. However, clinicians rarely use it when analyzing hemograms, possibly because physicians are not familiar with these meaningful parameters. Anisocytosis can be caused by inflammation, nutritional status, iron deficiency, inadequate erythropoietin production, or oxidative stress. It can lead to tiredness, shortness of breath, dizziness, headaches, cold hands and feet, pale skin, and chest pain [21]. We consider unlikely that the high RDW level and RDW/Lymphocytes index achalasia patients are due to anemia, because undergoing surgery typically recover their nutrition and gain weight; however, the anemia persists in the patients.

RDW appears to be a surrogate parameter of the inflammatory process, like the erythrocyte sedimentation rate, and more importantly, a surrogate parameter of fibrosis. In our view, the mechanism by which inflammation may increase RDW in patients with achalasia is through ineffective erythropoiesis mediated by cytokines, as has been demonstrated in the pathophysiology of other diseases where tumor necrosis factor alpha (TNF-α), interleukin (IL)-1β, IL-6, interferon gamma (IFN-γ), IL-17, and IL-22 impair erythrocyte maturation. These cytokines could modulate erythropoiesis through the following pathways: (i) inhibiting erythropoietin (EPO) gene transcription, (ii) blocking antiapoptotic and maturation effects of EPO, and (iii) decreasing renal EPO synthesis by inflammatory cytokine desensitization of EPO erythroid progenitors in bone marrow [22]. Furthermore, these cytokines directly inhibit the mean lifespan of erythrocytes and their membrane deformities. Therefore, inflammation may contribute to anisocytosis by damaging EPO-induced erythrocyte maturation and releasing immature erythrocytes into peripheral blood, thereby increasing RDW. In the sera of patients with achalasia, we observed increased levels of IFN-γ, IL-17, and IL-22 compared with HD [23], suggesting that this systemic inflammation could contribute to the increase in RDW.

In this study, RDW was positively correlated with ultrasensitive C-reactive protein (CRP) levels, and it predicted an increase in RDW levels in a multivariate analysis, independent of age, sex, and hemoglobin level (P=0.003). It was also determined that female patients had higher RDW levels, which were associated with a higher IRP. Simultaneously, linear analysis demonstrated the influence of sex and autoimmune disease on RDW alterations.

These results highlight the importance of RDW as a relevant marker in evaluating women with a diagnosis of achalasia and type II achalasia, showing significant variation over time. This finding clearly contrasts with the stability of the RDW observed in patients with GERD. The variation between baseline and follow-up RDW in achalasia suggests that RDW may reflect changes in disease activity and its natural evolution. It is essential to consider the characteristics and comorbidities of each patient.

The stability of RDW in patients with GERD may be due to differences in the pathophysiology between achalasia and GERD.

Furthermore, the association between RDW and IRP underscores the potential usefulness of RDW as an indicator of disease severity. Therefore, the positive correlation with the IRP suggests that RDW may be a complementary tool for assessing patients with achalasia. This relationship also indicates that alterations in RBCs associated with systemic inflammation may provide additional insights into the progression and prognosis of patients with achalasia.

Several hematological indices, such as NLR, MLR, RDW, and platelet-related markers, have been proposed as potential indicators of systemic inflammation [24]. Recently, the RDW/lymphocyte ratio has emerged as a prognostic biomarker in predicting the clinical course of various diseases, including cardiovascular diseases, sepsis, liver cirrhosis, autoimmune liver disease, cancers, leukemia, renal dysfunction, and respiratory disease. Moreover, it is a promising biomarker in positively predicting the severity of hepatic fibrosis and cirrhosis in non-alcoholic fatty liver disease patients [25-27]. Patients with achalasia types I and II had a significantly increased RDW/lymphocyte ratio compared with HD. However, at five years of follow-up in patients with type I achalasia, the RDW/lymphocyte ratio increases four times compared to type II achalasia (40% vs. 11%), suggesting that the former is associated to fibrous stage than the latter. This is consistent with what was previously reported by our group, where type I achalasia has the highest level of fibrosis compared with type II and type III achalasia [27]. The female group with achalasia exhibited a significantly higher RDW/lymphocyte ratio compared to the HD and GERD groups at the five-year follow-up. However, in male patients with achalasia, the RDW/lymphocyte ratio increases up to two times compared to female patients with achalasia (17% vs. 9%), at five years of follow-up, suggesting that the disease may be more aggressive in the latter.

Moreover, having a low-cost biomarker might enable the determination of low-grade inflammation, which has been demonstrated to contribute to the development of metabolic disorders (e.g., obesity, diabetes, and dyslipidemia) and an increased risk of chronic degenerative diseases, as well as a common pathogenic denominator in age-related diseases. This could influence the design of therapeutic strategies that enable long-term control of inflammation, thus preventing worse disease prognosis.

The advantages of using RDW are that it does not incur an additional charge and appears to be unaffected by infectious processes in the short term, unlike ESR and CRP levels.

However, limitations to consider when interpreting RDW include inter-study variability in laboratory methodologies, such as sample processing times and hematology analyzers, because the test is equipment- and laboratory-dependent. Each piece of equipment is calibrated with a different frequency histogram, and each laboratory defines its standard rate. HRM subtype classification lacks reported inter-observer reliability. Moreover, RDW can be altered by various medical conditions, including hepatic disease, anemia, and deficiencies in folate and vitamin B12 [26]. Other limitations merit consideration. The study was conducted at a single center, so incorporating samples from different research centers is necessary for the generalization and validation of our results. It is a retrospective study, which may introduce bias. The control group (healthy donors) is significantly younger, introducing selection bias. Moreover, an interesting and yet unanswered question exists as to whether the relationship between increased RDW and achalasia is causal or results from the underlying pathophysiological state. Due to the observational nature of the study, it does not allow for investigating the underlying mechanisms.

## Conclusions

RDW and RDW/lymphocyte ratio are noninvasive, routinely inexpensive, and straightforward clinical tests that can be performed in any laboratory setting. However, clinicians rarely use it when analyzing hemograms, possibly because they are not familiar with these meaningful parameters. RDW and the RDW/lymphocyte ratio appear to be surrogate markers of the inflammatory process, similar to the erythrocyte sedimentation rate. More importantly, they serve as surrogate parameters of fibrosis, which could prove to be a valuable tool for determining the progression of damage in the esophagus.

This study highlights the importance of RDW and the RDW/lymphocyte ratio as helpful markers for systemic silent inflammation in patients with achalasia, demonstrating notable temporal variation depending on sex (male) and type of achalasia (type I). Additionally, the positive correlation between RDW and IRP suggests its usefulness in clinical assessments as a valuable and easily determined tool for monitoring patients with achalasia. Moreover, we also determined that high levels of RDW and RDW/lymphocyte ratio could be associated with patients with type I achalasia, characterized by severe fibrosis of the esophageal muscle layer, compared with patients with type II achalasia. However, future prospective studies are required to confirm its role as a prognostic marker for disease severity in achalasia.
